# Balancing Therapy with Thrombopoietin Receptor Agonists and Splenectomy in Refractory Immune Thrombocytopenic Purpura: A Case of Postsplenectomy Thrombocytosis Requiring Plateletpheresis

**DOI:** 10.1155/2016/5403612

**Published:** 2016-10-12

**Authors:** Jacquelyn Zimmerman, Kelly J. Norsworthy, Robert Brodsky

**Affiliations:** Division of Hematology, Johns Hopkins University, Baltimore, MD, USA

## Abstract

Immune thrombocytopenic purpura (ITP) causes thrombocytopenia through the autoimmune destruction of platelets. Corticosteroids remain the first line of therapy, and traditionally splenectomy has been the second. While the availability of thrombopoietin receptor agonists (TPO-RAs) has expanded treatment options, there is little data for the ideal management of these agents in preparation for splenectomy. Thrombocytosis has been reported after splenectomy in patients treated with TPO-RA preoperatively, with one prior case requiring plateletpheresis for symptomatic thrombocytosis. We present a case report and review of the literature pertaining to this complication and provide recommendations for preventing postsplenectomy thrombocytosis in ITP patients on TPO-RAs.

## 1. Introduction

Immune thrombocytopenic purpura (ITP) in adults is characterized by the autoimmune destruction of platelets and an increased risk of bleeding [1, 2]. The hallmark of disease is thrombocytopenia that may be profound with associated bleeding, but the majority of patients achieve remission with appropriate treatment.

The mainstay of initial therapy remains corticosteroids, with 70–90% of patients responding to a steroid pulse [3–6]. However, response is frequently not maintained once steroids are tapered. Intravenous immunoglobulin (IVIG) is also used as first line of therapy in patients, often in combination with steroids when an accelerated platelet response is required. Splenectomy has traditionally been the recommended second line of therapy for patients without sustained response to steroids or IVIG, due to durable responses in up to 70% of patients [4, 7]. However, there are risks for immediate perioperative complications as well as long-term complications after splenectomy, including thrombosis, systemic infection with encapsulated organisms, and possible increase in risk of hematologic malignancy [7–9]. Thus, many hematologists prefer to try other medical therapies before pursuing such an invasive option [6, 7].

The availability of the well tolerated, nonimmunosuppressive thrombopoietin receptor agonists (TPO-RAs), eltrombopag and romiplostim, as second line treatment options for ITP has started to shift the paradigm of ITP management and delay splenectomy [7, 10–13]. In the subset of patients who do not achieve adequate responses to TPO-RAs, one must consider the optimal management of these agents prior to splenectomy. Here, we describe the case of a patient with highly refractory ITP who ultimately underwent splenectomy following therapy with romiplostim and suffered symptomatic thrombocytosis postoperatively, requiring plateletpheresis. We review three other cases in the literature reporting this complication and provide recommendations for preoperative management.

## 2. Case Report 

A 26-year-old woman with a history of rheumatoid arthritis presented to her rheumatologist in December 2013 with complaints of bruising and bleeding. The patient had noticed petechiae on her legs a month earlier, with progression to gingival bleeding with flossing. She also had a particularly heavy menstrual cycle three weeks prior to her presentation. Her rheumatoid arthritis disease modifying agents included weekly methotrexate and bimonthly adalimumab, both of which preceded her symptoms for more than one year. Her most recent adalimumab injection was one day prior to presentation and resulted in significant bruising, an adverse effect she had not previously experienced.

Physical exam was notable for petechiae over the left buccal mucosa and lower extremities, with diffuse ecchymoses worse on the lower extremities and the abdominal site of her adalimumab injection. She was sent to the emergency department, where she was found to have a platelet count of 1 × 10^9^/L and hemoglobin of 10 g/dL. She received a platelet transfusion without a response and underwent screening computed tomography (CT) scans of the head, chest, abdomen, and pelvis to rule out underlying bleeds, all of which were negative. Rheumatologic workup was negative and included normal studies for C3 and C4, haptoglobin, rheumatoid factor, and antiphospholipid antibodies. Other negative/unremarkable studies included platelet factor 4 antibody, D-dimer, fibrinogen, serum immunoglobulins, Epstein-Barr virus polymerase chain reaction, and thyroid function tests. Immature platelet fraction was elevated at 26.1%, consistent with destruction of platelets. Her peripheral smear demonstrated profound thrombocytopenia.

Our hematology service was consulted and felt that her presentation was most consistent with ITP. Secondary causes of ITP were ruled out with negative human immunodeficiency virus and hepatitis C screening. She was started on IVIG 0.5 gm/kg/day × 4 days as well as prednisone 1 mg/kg daily. On the day of discharge, four days later, her platelet count had increased to 15 × 10^9^/L.

Within one month of her initial diagnosis, she required readmission to the hospital for epistaxis/hemoptysis and a platelet count of 8 × 10^9^/L, despite steroids and IVIG. She was given the first of four weekly rituximab infusions at a dose of 375 mg/m^2^ and another dose of IVIG at 1 gm/kg, which augmented her platelet count to 52 × 10^9^/L. However, improvement in her platelet count was temporary, prompting three cycles of 40 mg dexamethasone for 4 days every 2 weeks, in combination with the rituximab, as described by Bussel et al. [14]. Given continued lack of response, a bone marrow biopsy was obtained and was unremarkable. After another drop in platelet count to 12 × 10^9^/L, eltrombopag was started. Her platelet counts stabilized, ranging from 30 to 90 × 10^9^/L, but the patient developed alopecia. As alopecia is a reported side effect of eltrombopag, it was weaned and she was transitioned to romiplostim. Suboptimal response to romiplostim ultimately led to a dose increase to 10 mcg/kg weekly, the maximum recommended dose. As platelet counts were still fluctuating significantly despite maximal medical therapy, with intermittent steroid requirements and IVIG, the patient ultimately underwent elective splenectomy approximately one year after her initial diagnosis. Her last dose of romiplostim was eight days prior to the procedure; her scheduled dose the day prior to surgery was held with concern for thrombosis risk. She received a dose of IVIG on the prior day to assure a platelet count above 50 × 10^9^/L for surgery. She did not receive preoperative corticosteroids at the preference of the surgical team. Her platelet count the day of surgery was 64 × 10^9^/L.

**Figure 1:**
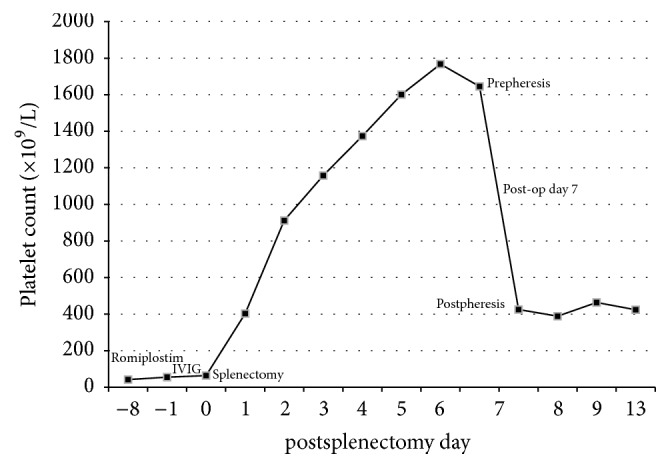
Platelet trend perisplenectomy and associated interventions. Patient received last dose of romiplostim eight days prior to surgery. At peak platelet levels, patient was symptomatic, prompting intervention with plateletpheresis.

Postoperatively, the patient was noted to develop thrombocytosis, beginning on postoperative day 1 and continuing until the day of discharge on postoperative day 5 (Figure 1). Given the extreme thrombocytosis, she was started on aspirin 325 mg daily. The following day, the patient followed up in hematology clinic for labs, and her platelet count was even higher at 1,767 × 10^9^/L. At this time, she developed symptoms of a headache, nausea, and dizziness, prompting admission to the hematology service for cytoreductive therapy with hydroxyurea and urgent plateletpheresis. Her postpheresis platelet count was 425 × 10^9^/L. Platelet counts remained stable after discharge, and hydroxyurea was discontinued without subsequent thrombocytosis. Most recent platelet count was 314 × 10^9^/L, and the patient is not on any dedicated ITP therapy.

## 3. Discussion

ITP management is challenging, as it is frequently difficult to achieve lasting platelet responses. Though the availability of TPO-RAs has diversified the arsenal for managing ITP, there is minimal precedent for incorporation into the management plan, specifically when splenectomy is pending. A recent retrospective study by Zaja et al. suggested that preoperative romiplostim or eltrombopag could improve operative bleeding risk by increasing platelet count in patients with chronic ITP refractory to steroids or IVIG, though most patients in this study notably received concurrent IVIG or corticosteroids with TPO-RA initiation [15]. There are two additional case reports describing the use of TPO-RAs to treat refractory thrombocytopenia perisplenectomy. Uwagawa et al. describe a case using eltrombopag to augment platelet counts prior to splenectomy [16]. Another case report describes recurrent thrombocytopenia after splenectomy after romiplostim dose was held in preparation for surgery; however, platelet count recovered once romiplostim was restarted [17]. As such, there may be a role for TPO-RAs in certain circumstances before splenectomy to increase platelet count to a safe surgical threshold.

However, given the known thrombotic complications with TPO-RAs [11, 18], one must also consider the postsplenectomy risk of thrombocytosis and thrombotic events, which typically warrant thrombosis prophylaxis and antiplatelet therapy [7, 19]. Postsplenectomy reactive thrombocytosis may occur in 75–85% of patients, and splenectomy accounts for 19% of extreme thrombocytosis [19, 20]. This risk is independent of concurrent or recent use of TPO-RAs, steroids, and IVIG.

A review of the MEDLINE database identified three additional case reports describing postsplenectomy thrombocytosis in patients with ITP receiving romiplostim before splenectomy (Table 1). Baldini et al. described a patient with transient thrombocytosis after using romiplostim to augment platelet count prior to splenectomy in a patient with ITP secondary to non-Hodgkin lymphoma [21]. Similarly, Sivera et al. reported a case of transient thrombocytosis after romiplostim was used to augment platelet count prior to splenectomy in a patient with ITP refractory to steroids and IVIG and with significant uterine bleeding [22].

There is only one prior case report of postsplenectomy thrombocytosis that required plateletpheresis. Raval et al. reported a patient who received a dose of romiplostim within 24 hours prior to splenectomy and subsequently developed extreme thrombocytosis requiring hydroxyurea and ultimately plateletpheresis due to headache, blurred vision, and nausea [23]. In this case, the patient experienced another peak in her platelet count to 1096 × 10^9^/L a few days later, requiring repeat of plateletpheresis for recurrent symptoms.

Romiplostim can be a challenging agent to manage, particularly due to its nonlinear pharmacokinetics. Overall, platelet counts appear to increase in a dose dependent manner; however, there is significant variability across patients, despite weight-based dosing [24]. Peak response to dosing occurs between 10 and 14 days after dose administration and also appears to have some dose dependence [24]. Based on the sustained half-life of romiplostim, Raval et al. recommend holding the drug 7–10 days prior to splenectomy [23]. For our patient, who was receiving 10 mcg/kg doses of romiplostim, peak platelet count would be predicted at 14 days after dosing or at postoperative day 6 when considering her last administered dose. There is also a role for drug elimination through binding to the TPO receptor on platelets and renal excretion, further complicating the pharmacokinetics [24]. Eltrombopag similarly demonstrates dose dependence, but with linear pharmacokinetics and an estimated half-life of 26–35 hours in patients with ITP [25]. These differences in pharmacokinetics may explain the lack of reported postsplenectomy thrombocytosis in patients receiving eltrombopag preoperatively.

In conclusion, there is minimal data to truly assess the temporal relationship between TPO-RA administration and extreme thrombocytosis after splenectomy in patients with ITP. While there is some data that TPO-RAs can be safe in the days leading up to splenectomy to achieve platelet numbers to minimize surgical risk, there is an inherent risk for postsplenectomy thrombocytosis that may be augmented by recent TPO-RA therapy. We would recommend avoiding romiplostim treatment for two weeks preoperatively, unless other medical measures have failed to raise the platelet count to safe levels. The concurrent use of IVIG and a TPO-RA in the perisplenectomy period may also increase thrombocytosis risk since the half-life of IVIG is prolonged in excess of two weeks. If TPO-RA therapy is required to boost the platelet count, we would advise against concomitant use with IVIG, given the risk for additive effects and the complication seen in our patient. Postoperatively, it is crucial to closely follow platelet counts, administer prophylaxis for thrombosis, and start an antiplatelet agent if necessary. Extreme cases with associated symptoms may warrant plateletpheresis to prevent thrombotic events.

## Figures and Tables

**Table 1:** Case reports of thrombocytosis following splenectomy for refractory ITP treated with TPO-RA preoperatively.

Patient	TPO receptor agonist	Platelets before splenectomy (×10^9^/L)	Maximum platelets after splenectomy (×10^9^/L)	Intervention	Postintervention platelet count (×10^9^/L)
Our case	Romiplostim 8 days earlier	55	1767	Apheresis hydroxyurea	425
Baldini et al. [21]	Romiplostim date not reported	55	740	None	198 (at 3 months)
Sivera et al. [22]	Romiplostim 5 days earlier	129	1013	None	Normalized at 2 months
Raval et al. [23]	Romiplostim 22 hours earlier	64	2058 1096	Apheresis × 2 hydroxyurea	522 324
